# Antioxidant Activity of Egg Yolk Protein Hydrolysates Obtained by Enzymatic and Sub-Critical Water Hydrolysis

**DOI:** 10.3390/molecules28237836

**Published:** 2023-11-29

**Authors:** Ismael Marcet, María Carpintero, Manuel Rendueles, Mario Díaz

**Affiliations:** Department of Chemical and Environmental Engineering, University of Oviedo, C/Julian Clavería 8, 33006 Oviedo, Spain; marcetismael@uniovi.es (I.M.); carpinteromaria@uniovi.es (M.C.); mariodiaz@uniovi.es (M.D.)

**Keywords:** sub-critical water hydrolysis, egg yolk, peptides, antioxidant capacity, enzymatic hydrolysis

## Abstract

Obtaining peptides with antioxidant properties by enzymatic hydrolysis has been widely described; however, the use of non-enzymatic methods to obtain peptides with antioxidant capacities has been poorly investigated. In this study, non-soluble proteins obtained from delipidated egg yolk granules were hydrolyzed with trypsin, and with a non-enzymatic method using sub-critical water hydrolysis under a non-oxidizing (nitrogen) and oxidizing (oxygen) atmosphere. The effect of the sub-critical water hydrolysis on the amino acids’ composition of the hydrolysates was assessed. Furthermore, the antioxidant capacities of the hydrolysates were evaluated using the ABTS^•+^ scavenging assay, the DPPH radical scavenging activity assay, and by measuring the reducing power of the peptides, the peptides’ ferrous ion chelating capacities, and the antioxidant effect of the peptides on beef homogenates. The hydrolysate obtained by sub-critical water hydrolysis under a nitrogen stream showed similar or better results in the antioxidant tests than those obtained using trypsin hydrolysis, except in the ferrous chelating capacity, where the trypsin hydrolysate showed the best performance. The oxidizing environment promoted by the oxygen in the other sub-critical water hydrolysis method tested produced the peptides with the lowest antioxidant capacities, due to changes in the primary structure of the peptides. These results suggest that the sub-critical water hydrolysis method under a nitrogen stream, in comparison with the enzymatic hydrolysis, is a reliable method to obtain peptides with good antioxidant capacities.

## 1. Introduction

In food preservation, it is important to keep oxidation processes under control. Lipid peroxidation is one of the most important causes of deterioration in precooked foods and meats, altering their organoleptic and nutritional properties [1]. In order to avoid these oxidation problems, the study of antioxidants has been widely investigated in recent years, and the use of additives, such as butylated hydroxyanisole, butylated hydroxytoluene and propyl gallate, has become widespread in the food industry. However, research into natural substances with similar or better antioxidant properties is interesting, and persists in the bibliography, and to this end, tea extracts, isoflavones, lycopene, vitamin C or peptides obtained from food proteins have been investigated for these features [2,3]. In particular, obtaining peptides with antioxidant properties is of particular interest due to their other potential bioactivities, such as antihypertensive [4] or cholesterol-lowering capacities [5]. Furthermore, the raw sources of these peptides can be proteins from food industry wastes, or proteins with limited functional properties recovered by using hydrolysis methods and revalued.

The antioxidant properties of these hydrolysates vary with the raw protein used and with the process selected to obtain the peptides. Therefore, many proteins have been investigated, such as soya protein, rapeseed, porcine hemoglobin, porcine myofibrillar protein or egg yolk and egg white proteins, among others. Concerning the methods used in obtaining the peptides, the most common are the enzymatic hydrolysis, microbial fermentation and chemical hydrolysis [6].

Among them, enzymatic hydrolysis is the most widely used strategy as it does not produce the by-products that usually occur during fermentation, does not produce chemical residues and has high selectivity and mild operating conditions [6,7]. Additionally, enzymatic hydrolysis has been frequently cited in the bibliography as a means of producing hydrolysates with interesting bioactive and antioxidant capacities [4,8,9,10].

Nevertheless, in the last year, there has been a growing interest in producing bioactive peptides through novel and green technologies, such as ultrasounds, microwaves, high hydrostatic pressure, pulse electric field and subcritical water hydrolysis (SWH) [6]. Regarding SWH, it has been proven to be a powerful tool to extract bioactive molecules and to obtain peptides from proteins difficult to hydrolyze. Water is sub-critical when the temperature is between 100 °C and 370 °C and the water is maintained in a liquid state by high pressure. In this state, noticeable changes occur in the physical–chemical properties of water. In this sense, at 250 °C, the ion concentration product (Kw) increases to 10^−11^ and the dielectric constant decreases from 80 at room temperature to 27, a value which is appropriate for dissolving compounds that are insoluble in liquid water at room temperature and at sea-level pressure [11,12]. In fact, sub-critical water has been used to recover useful compounds from biomass discards by many other researchers [13,14,15].

Regarding egg yolk, it can be easily separated to produce plasma and granular fractions. The plasma is mainly composed of lipids, while the granular fraction is protein-rich. In this regard, the plasma fraction shows similar features to those found in the whole egg yolk, with similar emulsion and gelation properties [16,17]. However, the granular fraction is formed by the interaction of different proteins through phosphocalcic bridges. This protein association is highly insoluble, and it requires an ionic strength ≥0.3 M NaCl to dissolve its protein constituent and, therefore, in solutions with low ionic strength, the functional properties of this fraction are reduced, and its range of applications is limited [18]. Consequently, the aim of this study is to analyze and compare the bioactive properties of peptides obtained from the hydrolysis of egg yolk granules using water in sub-critical conditions under an oxygen or nitrogen stream and an enzyme, trypsin. The antioxidant properties of the peptides obtained by SWH were tested in comparison with those of the peptides obtained by the enzymatic method (trypsin) in order to determine the potential of this non-enzymatic method for obtaining food-grade antioxidants.

## 2. Results and Discussion

### 2.1. Hydrolysate Characteristics

The characteristics of these hydrolysates were discussed in previous work [19]. In summary, in the SWH under nitrogen pressure, the reaction time was 240 min, and in the SWH under oxygen stream, it was 120 min. These times were necessary to solubilize 95% of the granular protein as soluble peptides in each case. The average sizes of the peptides were 4.1 ± 0.1 and 3.9 ± 0.2 kDa for SWH under a nitrogen and oxygen stream, respectively. In the case of enzymatic hydrolysis, 50% of the initial granular protein was recovered as soluble peptides, with an average size of 7.1 ± 0.3 kDa after 360 min of reaction.

The amino acid composition of the hydrolysates obtained and of the egg yolk granular fraction are shown in Table 1. As was expected, the amino acid composition of the proteins of the granular fraction and of the peptides obtained after the enzymatic hydrolysis of this fraction were quite similar. However, some composition differences with the granular protein were observed when the hydrolysates obtained from the sub-critical water hydrolysis were compared. In both nitrogen and oxygen sub-critical water hydrolysis, the increase in the amount of glycine, alanine and valine was noticeable. These amino acids were probably obtained, at least in part, from the hydrothermolysis of the serine [20], which decreased in concentration, as can be observed in Table 1. These results are in agreement with those obtained by other authors [21,22].

In the case of the sub-critical water hydrolysis under an oxygen stream, the amount of the simplest amino acids found was the highest, together with a greater decrease in other amino acids such as histidine, lysine and arginine. Furthermore, according to Stadtman et al. [23], the oxidation of proteins leads to the hydroxylation of aromatic groups and aliphatic amino acid side chains, nitrosylation of sulfhydryl groups and the chlorination of aromatic groups and primary amino groups among other alterations. Some of these changes in the primary structure of the peptides obtained cannot be observed in Table 1, since only the main amino acids were detected. Regarding the amount of free amino acids in the hydrolysates, these were detected by using the method of Rosen.

### 2.2. ABTS^•+^ Scavenging Assay

**Figure 1:**
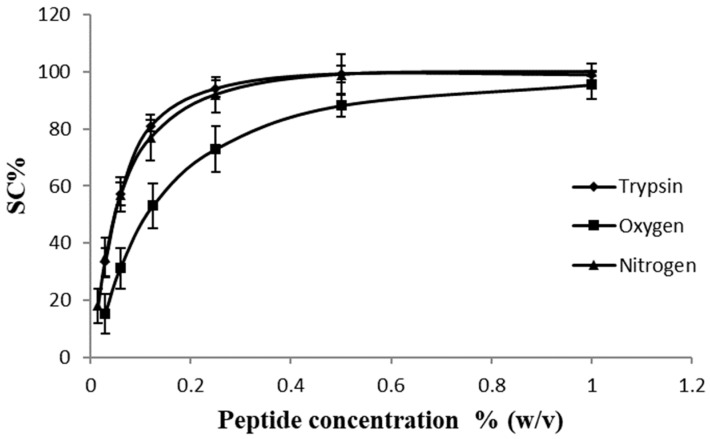
ABTS^•+^ scavenging capacity of the hydrolysate obtained by trypsin hydrolysis (diamond shape), SWH under nitrogen stream (triangle shape) and SWH under oxygen stream (square shape).

The ABTS^•+^ scavenging capacity of the hydrolysates at several peptide concentrations is shown in Figure 1. According to these results, the IC_50_ for the peptides obtained by SWH under a nitrogen stream was 0.5 ± 0.02 mg/mL; for the peptides obtained using enzymatic hydrolysis (trypsin), it was 0.6 ± 0.01 mg/mL; and in the case of the SWH under an oxygen stream, it was 1.1 ± 0.02 mg/mL. Aliaga et al. [24] and Zheng et al. [25] studied the mechanism of the antioxidant activity of various amino acids on the ABTS^•+^ radical and the effect of pH changes and reaction time, and they found that a hydrogen atom from a thiol, amino or hydroxyl group was necessary during the reaction. In this sense, according to Table 1, the primary structure of the peptides produced by SWH under an oxygen stream showed the lowest amount of histidine, lysine, arginine and tyrosine, which may have resulted in the lower ABTS^•+^ scavenging activity detected in comparison with those peptides produced by enzymatic hydrolysis and SWH under an inert gas stream (nitrogen).

### 2.3. DPPH Radical Scavenging Activity Assay

**Figure 2:**
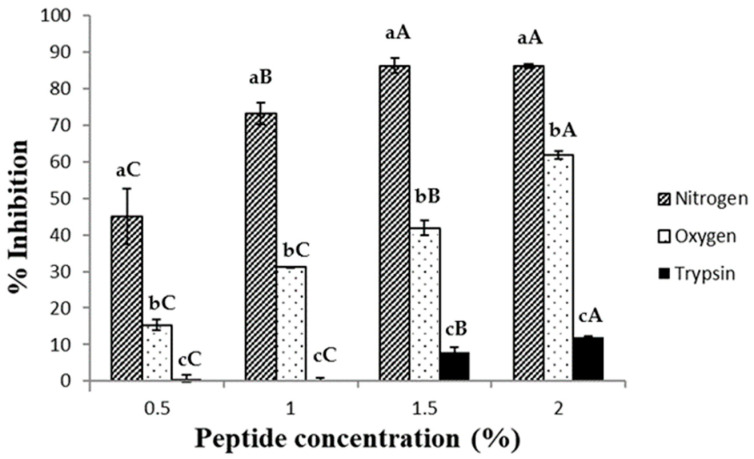
Inhibition of DPPH by the hydrolysates obtained using trypsin hydrolysis (black columns), SWH under nitrogen stream (hatched columns) and SWH under oxygen stream (dotted columns). Different letters between groups and concentrations indicate significant difference (*p* < 0.05).

DPPH is an organic compound frequently applied to evaluate the radical scavenging capacity of different compounds and extracts. In this case, the antioxidant substance acts as a proton-donor, producing the scavenging of the DPPH radical and decreasing absorbance at 517 nm [26]. In Figure 2, the inhibition of the DPPH radical by the peptides obtained by SWH and trypsin hydrolysis is shown. Peptides obtained using trypsin hydrolysis showed a very low antioxidant capacity compared with those obtained by the SWH method. This is because the ethanol medium decreased the solubility of these peptides, likely due to their large size in comparison with the peptides produced by SWH, preventing the quenching of the DPPH molecule [27]. However, peptides obtained with the SWH under a nitrogen stream showed the highest percentage of inhibition. The differences observed between the two SWH methods could be explained, as in the ABTS^•+^ scavenging section, by modifications in some amino acids under the oxygen pressure. DPPH scavenging values similar to those of the peptides produced by SWH under a nitrogen stream were observed by other authors, using egg yolk protein and enzymes [28]. These authors obtained around 90% DPPH inhibition using a mix of two enzymes, 6 h of hydrolysis (orientase, EC 3.4.21.62; and protease, EC 3.4.11.12) and an egg yolk protein hydrolysate concentration of 1%.

### 2.4. Peptide Reducing Power (RP)

**Figure 3:**
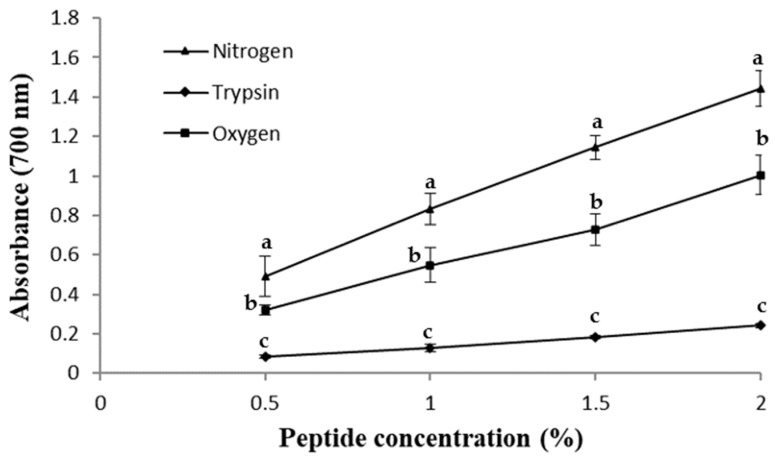
Reducing power of the peptides obtained by trypsin hydrolysis (diamond shape), SWH under nitrogen stream (triangle shape) and SWH under oxygen stream (square shape). Different letters between points at the same peptide concentration indicate significant difference (*p* < 0.05).

This method measures the capacity of the tested sample to reduce Fe^3+^-ferricyanide complex to the ferrous form (Fe^2+^) and, therefore, the antioxidant activity of the assessed compounds. The reducing power of the hydrolysates was calculated and is shown in Figure 3. In all cases, a correlation between the peptide concentration and the reducing power was observed, although this trend is less marked in the peptides obtained by trypsin hydrolysis. According to Chang et al. [29], there is a relation between the peptide size in hemoglobin hydrolysates and their reducing power. These authors suggested that the higher the average peptide size, the greater the capacity. However, in this case, this correlation was not confirmed, and the peptides obtained using enzymatic hydrolysis showed the lowest antioxidant activity. On the other hand, the smaller peptides obtained by the SWH method were seen to be significantly more effective in this test. He et al. [30] studied the trypsin hydrolysates of sarcoplasmic protein, myofibrillar protein and stromal protein from Paphia undulate, obtaining RP values of 0.58 ± 0.05, 0.57 ± 0.01 and 0.43 ± 0.01, respectively, using a peptide concentration of 1% (*w*/*v*). In this work, similar values were obtained in the SWH method under an oxygen stream (0.54 ± 0.09), and better results were obtained under a nitrogen stream (0.83 ± 0.08). However, using the same enzyme as these authors, the RP value was the lowest (0.13 ± 0.02). This could be due to the small size of the SWH peptides obtained, which enhances the interaction of the reducing amino acids and the substrate, increasing their RP.

### 2.5. Ferrous Ion Chelating Assay

**Figure 4:**
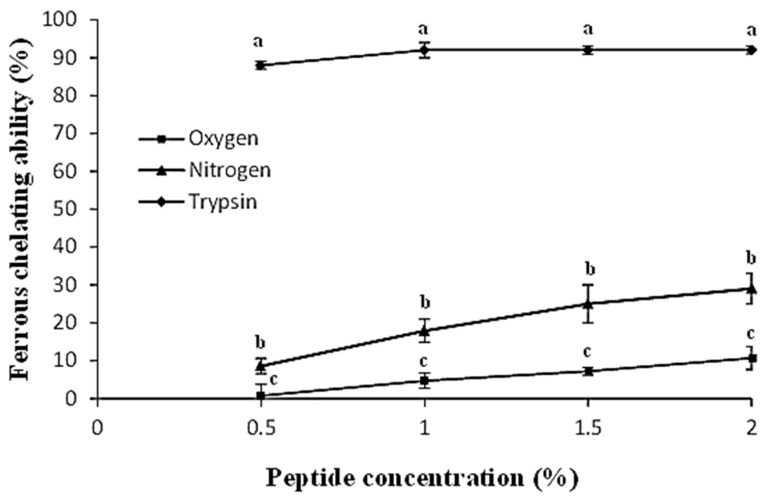
Ferrous chelating ability of the peptides obtained by trypsin hydrolysis (diamond shape), SWH under nitrogen stream (triangle shape) and SWH under oxygen stream (square shape). Different letters between points at the same peptide concentration indicate significant difference (*p* < 0.05).

In Figure 4, the chelating properties of the different hydrolysates are shown. According to other authors [31] the tryptic hydrolysates obtained from egg yolk protein have more chelating properties than those obtained from egg white protein. This is because one important protein present in the egg yolk is phosvitin. Phosvitin contain 135 residues of phosphoserine [32] and it is concentrated in the egg yolk granules. This is the most phosphorylated protein found in nature, and its chelating properties have been broadly studied [33,34]. Furthermore, the hydrolysates obtained from phosvitin maintain their chelating abilities [35]. In Figure 4, it can be observed how the tryptic hydrolysate of the egg yolk granules maintain high levels of chelating capacities even at the lowest peptide concentrations tested, probably due to the presence of phosvitin oligopeptides. According to Table 1, the high temperature and pressures necessary in SWH produced a decrease in the serine content of the hydrolysates, and, therefore, the dephosphorylation of the phosvitin and the reduction of the chelating capacities of the peptides obtained. Furthermore, other authors have detected a decrease in the ferrous chelating ability of the peptides obtained by the SWH method too. Specifically, Álvarez et al. [36] studied the chelating capacity of native hemoglobin in comparison to the hemoglobin hydrolysate obtained using SWH under a nitrogen stream, the latter showing a reduction in ferrous chelating ability. Other authors [29] have related peptide size with their chelating properties and concluded that peptides with a high molecular weight are more effective as ferrous chelating agents than those which are smaller. Thus, the lower size of the peptides obtained under nitrogen and oxygen streams, and the dephosphorylation of the phosvitin, may explain their limited ferrous chelating ability.

### 2.6. Antioxidant Effect of the Peptides in Beef Homogenates

**Figure 5:**
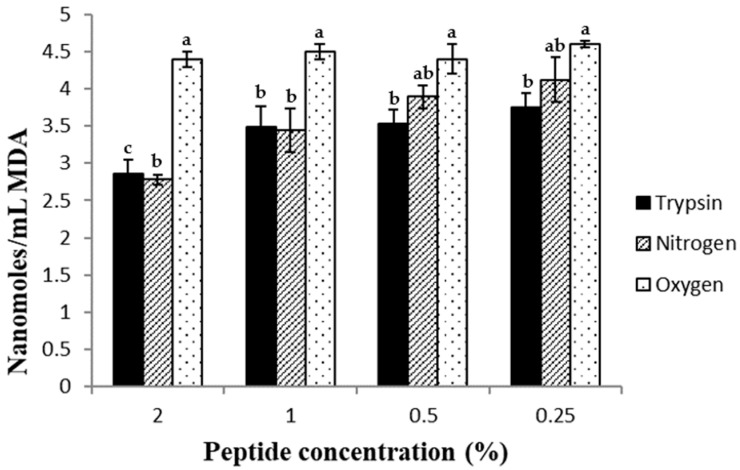
Effect of the hydrolysates on formation of TBARS in beef homogenates. Peptides obtained by trypsin hydrolysis (black columns), SWH under nitrogen stream (hatched columns) and SWH under oxygen stream (dotted columns). Different letters between columns at the same peptide concentration indicate significant difference (*p* < 0.05).

This test involves the generation of a pink chromophore measured at 532 nm due to the reaction between the thiobarbituric acid and the malondialdehyde produced during lipid oxidation. Other authors have related the antioxidant effect of some substances in beef homogenates to their capacity to chelate iron, due to the ability of these compounds to reduce the redox potential of the media [37,38]. For this reason, the peptides obtained by tryptic hydrolysis showed the best iron chelation capacity, as has been previously explained, and the best antioxidant effect in this assay (Figure 5). The peptides obtained by SWH under a nitrogen stream showed a similar antioxidant effect to those obtained by tryptic hydrolysis at high peptide concentration, although the ferrous chelating abilities of the former were less than those of the latter, as has been previously mentioned. However, at the lowest peptide concentration tested, slight differences were detected between the tryptic hydrolysate and that obtained by SWH under a nitrogen stream. These two hydrolysates showed noticeable differences in their ferrous chelating capacities, which did not correspond with the slight differences observed in their antioxidant effect on beef homogenates. For this reason, it is likely that the antioxidant effect, here, presented was due, at least in part, to another unknown mechanism.

Regarding the peptides obtained by the SWH method under oxygen stream, their antioxidant effect was undetected, showing values similar to those found in the control sample (4.3 ± 0.2).

### 2.7. Antimicrobial Test

Antimicrobial peptides obtained by enzymatic hydrolysis of hen egg white have been described by other authors [39,40,41,42]. Furthermore, the antimicrobial activity of hydrolyzed egg yolk proteins using digestive enzymes has been investigated too [31], although according to these authors, the antimicrobial activity of the hydrolysates was weaker and only appeared in those obtained using trypsin. However, in the present study, antimicrobial activity against *L. innocua* and *E. coli* could not be detected in any case, including that of trypsin, even when the highest peptide concentrations (40 mg/mL) were tested. These differences with the results described in the bibliography could be due to methodology and hydrolysate variations.

## 3. Materials and Methods

### 3.1. Delipidated Granules Obtention

The egg yolk granules were obtained according to Marcet et al. [43]. Egg yolk was separated from the albumen manually, and the vitelline membrane was discarded using tweezers. The egg yolk was diluted (1:1.5 *v*/*v*) with water and the pH was carefully adjusted to 7 by adding hydrochloric acid (0.1 M) with constant agitation in a mixer. Finally, the diluted egg yolk was kept overnight at 4 °C and centrifuged using a High Speed Refrigerated Centrifuge 6500 (Kubota, Japan) at 4 °C and 10,000 g for 45 min to obtain the granules in the sediment. Granules were lyophilized by using a Cryodos Lyophilizator (Telstar, Spain) and mixed with ethanol (96%, 1:12.5 *w*/*v*) at 40 °C for 2 h with gentle agitation. Finally, the granules were recovered using a vacuum pump and Whatman nr. 1 paper. The delipidated egg yolk granules were washed with 200 mL of fresh ethanol and dried overnight in a heater at 40 °C to obtain an ethanol-free powder.

### 3.2. Hydrolysates Production

The hydrolysates were obtained following the methodology describe and optimized by Marcet et al. [19]. Briefly, in the case of the sub-critical water hydrolysis method, the delipidated granules were dispersed in water (1:20 *w*/*v*) to obtain 400 mL of solution and placed in the jacketed reactor. Different temperatures were tested, and the best hydrolysis values were obtained at 180 °C. A non-reactive gas stream (nitrogen) or an oxidizing one (oxygen) were evaluated using a back-pressure valve to maintain the pressure at 40 bar. Furthermore, a gas stream of 1000 mL/min was selected and was controlled by a precision electrovalve (Brooks mass flow controller 5850). The injected gas was previously saturated with water to avoid sample evaporation and heated to keep the reactor temperature constant.

The enzymatic hydrolysis was carried out in a 400 mL bioreactor with a pH-STAT automatic titration (pH-Burette 24 2S, Crison, Spain) connected to an iso-thermal shaker. Delipidated egg yolk granules were mixed with water (1:20 *w*/*v*) and preheated at 90 °C for 7 min to enhance the dispersion of the granules. The enzyme–substrate ratio tested was 1/25, using trypsin from porcine pancreas E.C 3.4.21.4 (93610, Sigma-Aldrich) 18,136 U/mg. The dispersed granules were heated to 37 °C, and the pH was adjusted to 7.5 using 0.1 M sodium hydroxide solution. Finally, the enzyme was added to the reactor, and after 6 h, the enzymatic reaction was stopped by heating the hydrolysate for 15 min in a water bath at 90 °C. All the hydrolysates were frozen at −80 °C for at least 24 h and then freeze-dried.

### 3.3. Amino Acid Analyses

The amino acid profile of the peptides was analyzed by using a Jeol JLC-500/V amino acid analyzer (Jeol Ltd., Welwyn Garden City, UK) fitted with a Jeol Na^+^ high performance cation exchange column. For that purpose, lyophilized samples were previously hydrolyzed into free amino acids in a 6 M HCl solution at 110 °C for 23 h and then diluted 1 in 2 with the internal standard, norleucine, to give a final concentration of 125 μM.

### 3.4. ABTS^•+^ Scavenging Assay

ABTS^•+^ (2,2′-azinobis-(-3-ethylbenzothiazoline-6-sulfonic acid) radical scavenging capacity was calculated in each case according to a modified method described by Watchararuji et al. [44]. An initial stock solution of ABTS^•+^ composed of an aqueous solution of 7 mM ABTS^•+^, and 2.45 mM of potassium persulfate was prepared. This stock was kept in darkness at room temperature for 16 h before use. It was then diluted with distilled water until it reached an absorbance of 0.70 ± 0.04 at 734 nm in a 1 cm cuvette. Lyophilized samples were diluted at several concentrations (from 0.5 to 0.015% (*w*/*v*)), and aliquots of 166 μL were mixed with 4830 µL of the ABTS^•+^ solution and incubated in darkness at room temperature for 20 min. Finally, the absorbance of the samples was measured at 734 nm. Positive (GSH) and negative (water) controls were performed too. The percent of the scavenging capacity (SC%) at each concentration and the concentration necessary to decrease the absorbance to 50% of the initial value (IC_50_) were calculated. The SC% was calculated using the following equation:

SC% = [1 − (A_t_/A_r_)] × 100 (1)

where A_t_ is the absorbance of the sample and A_r_ is the absorbance of the negative control.

### 3.5. DPPH Radical Scavenging Activity Assay

The DPPH radical scavenging activity was determined as described by Marcet et al. [43] with slight modifications. The sample solution contained 0.6 mL of 1.0 mM DPPH radical solution, 0.6 mL of peptide solution at different concentrations (0.5–2% (*w*/*v*)) and 4 mL of ethanol (95%). The control solution contained 0.6 mL distilled water, 4.0 mL ethanol and 0.6 mL 1.0 mM DPPH. In all cases, the assay mixture was shaken vigorously using a mixer and incubated for 30 min in darkness at room temperature. After that, the absorbance was measured at 517 nm. In addition to the sample and the control solutions, the absorbance of the blank sample and the blank control were considered too. The results were calculated as the percentage inhibition according to the following formula:

% inhibition = [(A_sample-blank_ − A_control-blank_)/A_sample-blank_] × 100 (2)

### 3.6. Peptide Reducing Power (RP)

The reducing power of the hydrolysates was calculated according to the methodology described by Oyaizu [45] with slight modifications. Lyophilized peptides were dissolved in phosphate buffer (pH 6.6, 0.2 M) at several protein concentrations, and 4 mL of this test sample was mixed with 2 mL of 1% (*w*/*v*) potassium ferric cyanide. The mix was maintained at 50 °C for 20 min and cooled quickly. After that, 2 mL of trichloroacetic acid (10% *w*/*v*) was added, and the solution was centrifuged at 10,000× *g* for 10 min. Afterwards, the sediment was discarded, and 2 mL of the supernatant was mixed with 2 mL of water and 2 mL of ferric chloride (0.1% *w*/*v*). Finally, after 10 min the absorbance was measured at 700 nm. A high absorbance was indicative of strong reducing power.

### 3.7. Ferrous Ion Chelating Assay

The ferrous ion chelating ability of the peptides was assayed according to the method of Decker et al. [46]. Briefly, 5 mL of the sample solutions was mixed with 0.1 mL of 2 mM FeCl_2_ and 0.2 mL of 5 mM ferrozine solution. After incubation at room temperature for 10 min, the absorbance was measured at 562 nm. The complex Fe^2+^/ferrozine has a high absorbance at this wavelength, so high chelating ability is shown as a low absorbance. The chelating ability in percentage was calculated as follows:

Ferrous chelating ability (%) = (A_blank_ − A_t_/A_t_) × 100 (3)

where $A_{t}$ is the absorbance of the test sample.

### 3.8. Antioxidant Effect of the Peptides on Beef Homogenates

Minced ground beef (20% fat) was homogenized (20% *w*/*v*) in a 50 mM HEPES buffer solution at pH 7 using a Grindomix homogenizer (model GM300, Retsch, Germany). For that purpose, buffered systems have been widely used to study oxidation reduction reactions in meat systems [37]. The test solution contained 0.8 mL of the meat homogenate and 0.2 mL of distilled water or the peptide solution. These assay solutions were incubated at 37 °C for 60 min to then be tested in the TBARS formation. Butylated hydroxytoluene (20 μL, 0.2%) was added to the reaction mixture after the incubation to stop the oxidation.

A 0.25 M HCl solution containing trichloroacetic acid (TCA) 15% (*w*/*v*) and 2-thiobarbituric acid (TBA) 0.375% (*w*/*v*) was prepared using agitation and mild temperature to dissolve the components. Then, 2 mL of this HCl solution was added to 1 mL of the incubated test mixture and heated for 10 min in a boiling water-bath. The solutions were cooled at room temperature and centrifuged for 20 min at 5000× *g*. Finally, the resulting pink color was measured at 532 nm. TBARS were calculated from a standard curve of malonaldehyde (MDA), a breakdown product of tetraethoxypropane.

### 3.9. Antimicrobial Test

Two species, *Listeria innocua* (Gram positive) and *Escherichia coli* (Gram negative), were selected to test the antimicrobial effect of the peptides. They were grown in BHI and NB liquid medium at 37 °C for 24 h, respectively. The final bacterial concentration was diluted to 1 × 10^7^ UFC/mL, and 200 μL of these bacterial solutions were spread over the surface of a BHI and NB agar plate. In this agar medium, three wells were made in each plate using a sterile punch, and 50 μL of the test sample were placed in each one. The peptide concentration assayed in each case was from 2 to 40 mg/mL. The plates were incubated for 24 h at 37 °C, and after that, the inhibition zone was measured. A negative control was performed using distilled water as well.

### 3.10. Statistical Analysis

The assays showed in this work were carried out in triplicate. Mean and standard deviation are provided for each case. To assess the statistical differences between the methods used to obtain the hydrolysates, an analysis of variance (ANOVA) with a confidence level of 95% was applied, employing the Fisher’s test to determine the least significant differences (LSD) between samples. To carry out these statistical analyses, the Statgraphics Centurion XVI software (version 16.1.11) was used.

## 4. Conclusions

The antioxidant properties of the hydrolysates obtained using SWH under a nitrogen stream were found to be statistically greater than those found in the hydrolysates obtained using trypsin. Only in the ferrous chelating capacity was enzymatic hydrolysis seen to be a better choice, and this could be mainly explained by the dephosphorylation of the phosvitin during the SWH. In the other antioxidant tests, the differences observed could be explained by the smaller size of the SWH hydrolysates.

The SWH under an oxygen stream is the fastest of the methods used to obtain peptides from insoluble egg yolk proteins. However, its antioxidant capacities were significantly decreased in comparison with the other two methods, probably because of variations in the primary structure of the peptides. Therefore, it can be concluded that the SWH under a nitrogen stream is a non-enzymatic and non-aggressive method to be considered for the production of peptides with antioxidant properties, which could be employed in the food industry. Nevertheless, it should be taken into account that the harsh reaction conditions could produce undesired byproducts that would require further study.

## Figures and Tables

**Table 1:** Amino acid composition (mol%) of the hydrolytes obtained and of the egg yolk granules.

Amino Acid	Granules	Enzyme Hydrolysis	Sub-Critical Water Hydrolysis	
		Trypsin	Nitrogen	Oxygen
**Gly**	5.4	5.3	9	13
**Thre**	4.5	4.5	2.6	4
**Ser**	12	11.8	4.4	6.3
**Cys**	1.6	1.6	0.5	2
**Tyr**	2.8	2.7	2.9	0.6
**Asp**	9.1	9.2	3.7	6
**Glu**	11.3	12	14.5	21
**Ala**	7.2	7	12.3	12
**Met**	2	2	2.8	1.2
**Val**	6.1	5.9	8	9.6
**Ile**	5	4.9	5.2	4.7
**Leu**	7.9	8	10	7.5
**Pro**	5	4.9	6	1.2
**Phe**	2.8	2.7	3.4	1.3
**His**	2.6	2.5	2.8	1.1
**Lys**	7.4	7.2	5.8	3.5
**Arg**	7	7	6.2	4.8

## Data Availability

Data is contained within the article.
